# Quinolone and Cephalosporin Resistance in Enteric Fever

**DOI:** 10.4103/0974-777X.68529

**Published:** 2010

**Authors:** Malini Rajinder Capoor, Deepthi Nair

**Affiliations:** *Department of Microbiology, Vardhman Mahaveer Medical College and Safdarjung Hospital, New Delhi, India*

**Keywords:** Azithromycin, Ciprofloxacin resistance, Enteric fever, Molecular targets, Penems, Tigecycline

## Abstract

Enteric fever is a major public health problem in developing countries. Ciprofloxacin resistance has now become a norm in the Indian subcontinent. Novel molecular substitutions may become frequent in future owing to selective pressures exerted by the irrational use of ciprofloxacin in human and veterinary therapeutics, in a population endemic with nalidixic acid-resistant strains. The therapeutics of ciprofloxacin-resistant enteric fever narrows down to third- and fourth-generation cephalosporins, azithromycin, tigecycline and penems. The first-line antimicrobials ampicillin, chloramphenicol and co-trimoxazole need to be rolled back. Antimicrobial surveillance coupled with molecular analysis of fluoroquinolone resistance is warranted for reconfirming novel and established molecular patterns for therapeutic reappraisal and for novel-drug targets. This review explores the antimicrobial resistance and its molecular mechanisms, as well as novel drugs in the therapy of enteric fever.

## INTRODUCTION

Enteric fever remains a major public health problem in developing countries. The estimated incidence is approximately 33 million cases each year. In the developed countries, the incidence is much lower, and most cases are usually from travelers returning from endemic areas. With the development of multi-drug resistance (resistance to ampicillin, chloramphenicol, co-trimoxazole) in *Salmonella enterica* serovar Typhi and Paratyphi A in the 1990s, ciprofloxacin had been introduced as the first-line therapy. However, these strains, with decreased susceptibility to ciprofloxacin causing treatment failure, have become endemic in several countries in the Indian subcontinent.[1–3] Furthermore, high-level ciprofloxacin resistance has become common in this region. The therapeutics of ciprofloxacin-resistant enteric fever narrows down to third- and fourth-generation cephalosporins, azithromycin, tigecycline and penems.[4–8] In this communication, we explore newer antimicrobials and molecular targets in enteric fever isolates.

## ENTERIC FEVER: HISTORY

*Typhoid fever, or enteric fever, is a* major human infectious disease since centuries, surviving in conditions of poor sanitation, crowding and social chaos. It was responsible for the Great Plague of Athens at the end of the Peloponnesian war. The name *Salmonella Typhi* is derived from the ancient Greek *typhos*, an ethereal smoke or cloud that was believed to cause disease and madness. In the advanced stages of typhoid fever, the patient’s consciousness becomes extremely clouded. Although antibiotics have markedly reduced the frequency of enteric fever in the developed world, it remains endemic and a major public health infection in developing countries.[1]

## TRADITIONAL ANTIMICROBIALS: ENTERIC FEVER

Antibiotics that have been traditionally incorporated in the therapy of enteric fever are ampicillin, chloramphenicol, sulfamethoxazole-trimethoprim and tetracycline. With initial reports of chloramphenicol resistance in Mexico and the Indian subcontinent in the 1970s, in the year 1989, outbreaks due to plasmid, R-type ACCoSuTTm of H_1_ incompatibility group were reported worldwide. This led to the use of quinolones as the first line of therapy for typhoid fever in the 1990s.[2]

## ENTERIC FEVER: EMERGENCE OF NALIDIXIC-ACID-RESISTANT *S.*TYPHI (NARST) AND HIGH-LEVEL CIPROFLOXACIN RESISTANCE

Subsequently, NARST, with decreased susceptibility to ciprofloxacin (0.125-1 μg/L) causing therapeutic failure, emerged worldwide and became endemic in the Indian subcontinent.[3] Consequently, high-level ciprofloxacin-resistant enteric fever evolved in the Asian countries, including India.[3–10] Contrary to this, a high-level fluoroquinolone resistance in non-enteric fever salmonellae was reported frequently; with minimum inhibitory concentration (MIC) ranging from 16 to 64 μg/mL.[11]

The quinolones used in therapy of enteric fever are ciprofloxacin, gatifloxacin, levofloxacin and ofloxacin. In a previous study done on 31 ciprofloxacin-resistant isolates of enteric fever, on E-strip MIC testing, all the isolates showed an MIC ≥ 32 μg/mL.[5] In prior studies from India and Nepal, first- and second-generation quinolones had varying results. Gatifloxacin demonstrated better *in vitro* activity compared to other quinolones.[5,10,12] It was concluded that all fluoroquinolones should be tested individually, and ciprofloxacin does not represent this group adequately. In gatifloxacin and moxifloxacin, the primary target is *GyrA* gene; and for ciprofloxacin and levofloxacin, it is *ParC* gene. This would explain their varied pattern of susceptibilities. Disparity in the MIC levels of quinolones is attributable to difference in the additional fluoro- and other substitutions in their chemical structure.[5]

## USE OF EXPANDED-SPECTRUM CEPHALOSPORINS

Expanded-spectrum cephalosporins, such as cefipime, cefpodoxime proxetil, ceftriaxone and cefixime, have shown promise as therapies for the treatment of enteric fever. However, only cefixime and cefpodoxime proxetil have oral route of administration, while ceftriaxone and cefipime have parenteral route. Also, cefpodoxime proxetil has a favorable pharmacokinetic profile, which allows twice-daily administration. In a prior study from the authors found that all the 50 strains were sensitive to ceftriaxone, cefixime, cefpodoxime.[13] Cefixime and cefpodoxime are oral, but the cost of cefpodoxime is less than that of cefixime. Resistance is also emerging to extended-spectrum cephalosporins: ceftriaxone, cefixime, cefipime.[3,14,15] These alternative regimens have several disadvantages: their high costs, intravenous route of administration and prolonged defervescence time.[7] In another study amongst the cephalosporins tested, cefotaxime, a parenteral third-generation cephalosporin, demonstrated better results as it had the least MIC50 and MIC90, compared to oral third-generation cefixime and parenteral fourth-generation cefipime.[14]

Cefixime has gained popularity in prescription in India since the quinolones showed therapeutic failure. This could be the reason for their rising MIC levels. Cefipime had the lowest MIC90, 0.25 μg/mL, for *S.* Typhi and *S.* Paratyphi A, which could be attributed to its parenteral route of administration, making it less popular than cefixime. Only a single isolate of *S.* Typhi was an extended-spectrum β-lactamase (ESBL) producer and therefore had a high MIC for cephalosporins. Until now, there are a few reports of ESBL producers in *S.* Typhi and *S.* Paratyphi A.[12] There are many reports of ESBL producing *S.* Typhimurium attributed to plasmid-mediated class A ESBLs belonging to the TEM, SHV and CTX-M or PER CMY family.[11,14] This type of resistance being transferable, the major risk would be its transfer to *S.* Typhi and *S.* Paratyphi A. The reports of their rising MIC[3,15] are alarming; their overuse in outpatient settings can induce and select strains with ESBLs, early reports of which are emerging from the Indian subcontinent.[12–14]

## NEWER DRUGS FOR TYPHOID FEVER

### Azithromycin

Azithromycin, a broad-spectrum azilide, has prolonged intracellular concentrations and half-life. A large number of experimental and clinical trials done in the 1990s support its efficacy in traditional multi-drug-resistant typhoid fever.[16–18] However, the studies reporting the MIC of azithromycin and newer quinolones in the current scenario of ciprofloxacin-resistant enteric fever are scarce.[5,19–21] A rise in MIC over the years[5,16–19] has been attributed to irrational prescription for minor community-acquired upper respiratory, ear and sinus infections in the last decade. The misuse of azithromycin has been propelled due to its oral route of administration, as well as broad-spectrum antimicrobial activity with minimal side effects and interactions. In enteric fever, its role needs to be appreciated, as it is very effective in removing intracellular salmonellae, defervescence is rapid, gastrointestinal carriage is eradicated and, in particular, it represents a potential alternative in the pediatric population for whom quinolones are contraindicated.[17] *In vitro*, azithromycin has an MIC range of 4-16 μg/mL against *S.* Typhi. Higher clinical and bacteriological cure rate is attributable to >100-fold intracellular concentrations of azithromycin in macrophages as compared to serum.[17] The intracellular MIC may not be represented fully by currently available *in vitro* MIC testing methods, and such testing should be coupled with therapeutic trials. Due to its negligible relapse rate, fecal carriage, favorable outpatient compliance, azithromycin could become the preferred drug of choice over ceftriaxone, ofloxacin and chloramphenicol.[16,21]

### Tigecycline

Tigecycline is a glycylcycline (tetracycline analogue). It inhibits protein synthesis and evades efflux and target-mediated resistance to classical tetracyclines.*In vivo* and *in vitro* studies have demonstrated acquired resistance associated with up-regulation of chromosomally mediated efflux pump.[22,23] Tigecycline lacks cross-resistance with other compounds; it could aid in therapy of pan-drug-resistant salmonelloses. Nevertheless, systematic large-scale *in vivo* studies are needed to assess the relative merits of tigecycline versus other drugs in these infections. In prior studies, tigecycline was found to be very potent, inhibiting 97.3% of *S.* Typhi and all the *S.* Paratyphi A and ceftriaxone-resistant *Salmonella* isolates.[14,24,25]

### Carbapenems

The penems are a class of β-lactam antibiotics with broad-spectrum activity and are stable to hydrolysis by extended-spectrum β-lactamases-producing isolates.[26] In a recent study, the MIC90 for the carbapenems imipenem and meropenem in *S.* Typhi and *S.* Paratyphi A (0.064 μg/mL each) was less. Overall, faropenem had higher MIC90 at 0.25 μg/mL.[14] Prior reports have observed faropenem to be less active than imipenem; MIC90 for was 0.5 to 1 μg/mL.[27,28]

Although the use of azithromycin, tigecycline and carbapenem is not recommended by Clinical Laboratory Standards Institute,[29] yet it may become crucial, especially in the setting of ciprofloxacin-resistant and extended-spectrum β-lactamase-producing salmonellae in enteric fever.[12] Moreover, with increase in incidence of unusual and complicated paratyphoid fever,[10,11,28] newer broad-spectrum drugs need to be explored in the scenario of pan-drug-resistant salmonellae. Meanwhile, clinical efficacy trials are warranted to reach a definite conclusion in this regard.

## ROLLBACK OF SENSITIVITY TO THE TRADITIONAL DRUGS: FULL CIRCLE

In majority of recent Indian studies, a rollback of sensitivity to the classical first-line agents has been observed due to their restricted use in the 1990s.[3,9,10] In a recent Indian study, multi-drug resistance to anpicillin, chloramphenicol, co-trimoxazole (ACCO) was observed in 4% to 7% of isolates.[3,14] Nevertheless, re-introduction of chloramphenicol in enteric fever therapeutics has a long way ahead.

## MOLECULAR BASIS OF RESISTANCE OF QUINOLONES IN ENTERIC FEVER

Emergence of nalidixic-acid–resistant *S.* Typhi (NARST) with reduced susceptibility to ciprofloxacin was mediated by a single-nucleotide polymorphism (SNP) in quinolone resistance-determining region (QRDR) of *gyrA* at Ser83 or 87 Asp. Resistant isolates harbor two or more mutations in *gyrA, gyrB,* topoisomerase (*parC* and *parE*). Other mechanisms demonstrated are efflux pumps associated with multi-antibiotic resistance (MAR locus, outer membrane proteins), qnr plasmid (qnr A, qnr A, AAC1F) and up/down-regulation of operon genes.[30–32] Experimental evidence from *in vitro* selection studies suggests that single mutations are associated with low-level fluoroquinolone resistance, and high-level resistance is built up by sequential accumulation or perhaps a mixture of target and efflux-related mutations.[33] These are well documented in non-enteric fever salmonellae and other organisms,[34–36] as each target-gene mutation reduces the susceptibility by 4-8-fold.[37] A recent report observed that for *S.* Typhi, nalidixic acid resistance does not completely predict decreased ciprofloxacin susceptibility.[38] All the more, the emergence of plasmid-mediated quinolone resistance (PMQR) mediated by QNR, aminoglycoside acetyltransferase (AAC) and Qep A in family Enterobacteriaceae has complicated the understanding of molecular mechanisms of quinolone resistance.[39] Recent literature cites reports of molecular analysis of high-level ciprofloxacin resistance in enteric fever, worldwide, mainly in the Indian subcontinent.[7,8,31,40,43]

Single 83 Ser→Phe, 87 Asp→Asn, 72 Phe→Tyr substitutions are commonly associated with NARST.[32,36,42] Substitution at 133Glu→ Gly has not been observed previously in *S.* Typhi, *S.* Paratyphi A, other salmonellae or *E. coli.*[30,32,36,40,44,45] The mutations at 76 and 72 positions are also infrequently reported; nonetheless, there are few citations of single substitution at 76 Asp→Asn and 72 Phe→Tyr in *S.* Typhi [42] and 72 Phe→Tyr in combination with 83 Ser→Phe in *S.* Senftenfberg.[34]

However, In the studies, the genes encoding qnr plasmid protect[42,45] (qnr A, qnr B, AAC1-F) were not detected in ciprofloxacin-resistant or decreased-susceptibility strains. These proteins protect the target enzymes (DNA gyrase and type IV topoisomerase) from quinolone inhibition, and the AAC enzyme acetylates quinolones. Although these PMQR determinants confer only low-level resistance, nonetheless, they provide a background in which selection of additional chromosomal encoded quinolone-resistance mechanisms occurs.[46] These may become important in future in *S.* Typhi and *S.* Paratyphi A, as linkage between qnr plasmid, genes encoding extended-spectrum β-lactamases and AmpC type β-lactamases may reflect association between resistance to quinolones and extended-spectrum cephalosporins.[39,47]

## CONCLUSION

A large number of epidemiological and molecular studies are warranted to know novel target genes, thereby aiding in new drug-discoveries. Novel substitutions may become frequent in future owing to selective pressures exerted by the irrational use of ciprofloxacin in human and veterinary therapeutics, in a population endemic with NARST strains. The therapeutics of ciprofloxacin-resistant enteric fever narrows down to third- and fourth-generation cephalosporins, azithromycin, tigecycline and penems, which are unaffordable in nations with limited resources. Of the first-line antimicrobials, ampicillin, chloramphenicol and co-trimoxazole, especially chloramphenicol, need to be rolled back. Therefore, antimicrobial surveillance, coupled with molecular analysis of fluoroquinolone resistance, is warranted for reconfirming novel and established molecular patterns for therapeutic reappraisal and for novel drug targets.
